# Expression of Heterologous Cellulases in *Thermotoga* sp. Strain RQ2

**DOI:** 10.1155/2015/304523

**Published:** 2015-07-26

**Authors:** Hui Xu, Dongmei Han, Zhaohui Xu

**Affiliations:** Department of Biological Sciences, Bowling Green State University, Bowling Green, OH 43403, USA

## Abstract

The ability of* Thermotoga *spp. to degrade cellulose is limited due to a lack of exoglucanases. To address this deficiency, cellulase genes Csac_1076 (*celA*) and Csac_1078 (*celB*) from* Caldicellulosiruptor saccharolyticus *were cloned into* T. *sp. strain RQ2 for heterologous overexpression. Coding regions of Csac_1076 and Csac_1078 were fused to the signal peptide of TM1840 (*amyA*) and TM0070 (*xynB*), resulting in three chimeric enzymes, namely, TM1840-Csac_1078, TM0070-Csac_1078, and TM0070-Csac_1076, which were carried by* Thermotoga-E. coli *shuttle vectors pHX02, pHX04, and pHX07, respectively. All three recombinant enzymes were successfully expressed in* E. coli *DH5*α* and* T. *sp. strain RQ2, rendering the hosts with increased endo- and/or exoglucanase activities. In* E. coli*, the recombinant enzymes were mainly bound to the bacterial cells, whereas in* T. *sp. strain RQ2, about half of the enzyme activities were observed in the culture supernatants. However, the cellulase activities were lost in* T. *sp. strain RQ2 after three consecutive transfers. Nevertheless, this is the first time heterologous genes bigger than 1 kb (up to 5.3 kb in this study) have ever been expressed in* Thermotoga*, demonstrating the feasibility of using engineered* Thermotoga *spp. for efficient cellulose utilization.

## 1. Introduction

Due to rising global energy demands, developing renewable forms of energy, such as solar, hydro-, and bioenergy, has become increasingly important. Traditionally, bioenergy is generated by fermenting the glucose derived from starch. Starch alone, however, accounts for too small a fraction of biomass to sustain a positive energy balance. The need to replace fossil fuels demands that cellulose, the most common and renewable organic material on Earth [1], must not be overlooked. Cellulose is a linear polymer of D-glucose units linked by 1,4-*β*-D-glycosidic bonds. It becomes useful as a food and energy source once it is broken down into soluble cellobiose (*β*-1,4 glucose dimer) and glucose, a process called hydrolysis because a water molecule is incorporated for each dissociated glycosidic bond. Effective hydrolysis of cellulose requires the cooperation of three enzymes, namely, endo-1,4-*β*-glucanase (EC 3.2.1.4), exo-1,4-*β*-glucanase (also called cellobiohydrolase) (EC 3.2.1.91), and *β*-glucosidase (EC 3.2.1.21) [2–5]. Endoglucanase randomly breaks down the *β*-1,4 linkages in the regions of low crystallinity, exoglucanase removes cellobiose units from the nonreducing ends of cellulose chains, and *β*-glucosidase converts cellobiose into glucose. In general, exoglucanases degrade cellulose more efficiently than endoglucanases.

Hyperthermophilic bacteria* Thermotoga* are attractive candidates for the production of biohydrogen and thermostable enzymes. Surveying of* Thermotoga* genomes in the CAZy database (http://www.cazy.org/) revealed dozens of carbohydrate-active enzymes, the molecular foundation that allows* Thermotoga* strains growing on a wide range of carbon sources such as glucose, xylose, semicellulose, starch, and carboxymethyl cellulose (CMC) [6–9]. Nevertheless, the apparent absence of exoglucanases suggests the limited ability of these organisms to use cellulose as their main carbon and energy source. Up to date, there are only two reports describing low levels of exoglucanase activities in* Thermotoga* [2, 4], a phenomenon probably caused by nonspecific reactions of endoglucanases [10–12].

This study aimed at introducing heterologous exoglucanase activities into* Thermotoga* by genetic engineering.* T*. sp. strain RQ2 was selected as the host strain, because its genome encodes the largest repertoire of carbohydrate-active enzymes among all published* Thermotoga* genomes (Table 1). Moreover,* T.* sp. strain RQ2 has recently been discovered to be naturally transformable, making the transformation procedure straightforward [13]. The selection of candidate cellulases was focused on* Caldicellulosiruptor saccharolyticus*, a Gram-positive anaerobe growing optimally at 70°C and can use cellulose as a sole carbon source [14]. Csac_1076 (CelA) [15, 16] and Csac_1078 (CelB) [17, 18] of* C. saccharolyticus* DSM 8903 have been experimentally characterized as multidomain proteins with both endo- and exoglucanase activities and are suitable candidates to be introduced into* T. *sp. strain RQ2. However,* Caldicellulosiruptor* are Gram-positives and* Thermotoga* are Gram-negatives; CelA and CelB are unlikely to be secreted properly in* T. *sp. strain RQ2. The* Thermotoga* host would not benefit much from the heterologous cellulases unless the enzymes can be secreted to the extracellular environment. Signal peptides with a* Thermotoga* origin would be required to guide the transportation of foreign proteins in* T. *sp. strain RQ2. A literature search revealed that* T. maritima* TM1840 (amylase A, AmyA) [19–21] and TM0070 (xylanase B, XynB) [20] have been experimentally confirmed to be secretive proteins. The former is anchored on the “toga” part with catalytic domain facing outward, and the latter is secreted into the environment after the cleavage of its signal peptide. Therefore, the promoter regions and the signal peptide sequences of TM1840 (*amyA*) [19–21] and TM0070 (*xynB*) [20] were chosen to control the expression and transportation of the* Caldicellulosiruptor* cellulases in* T.* sp. strain RQ2.

## 2. Materials and Methods

### 2.1. Strains and Cultivation Conditions

The bacterial strains and vectors used in this study are summarized in Table 2. All* E. coli *strains were cultivated in Luria-Bertani (LB) medium (1% tryptone, 1% NaCl, 0.5% yeast extract) at 37°C.* Thermotoga* strains were cultivated at 77°C, 125 rpm in SVO medium [22]. SVO plates were made with 0.25% (w/v) gelrite [23].* Thermotoga* plates were put into Vacu-Quik Jars (Almore International Inc., Portland, OR, USA) filled with 96 : 4 N_2_-H_2_ and 4 g palladium catalyst (to remove oxygen) and incubated at 77°C for 48 h. When needed, ampicillin was supplemented into LB medium to a final concentration of 100 *μ*g mL^−1^, and kanamycin was added into liquid SVO medium and SVO plates to a final concentration of 150 and 250 *μ*g mL^−1^, respectively.

### 2.2. Construction of Vectors

**Figure 1:**
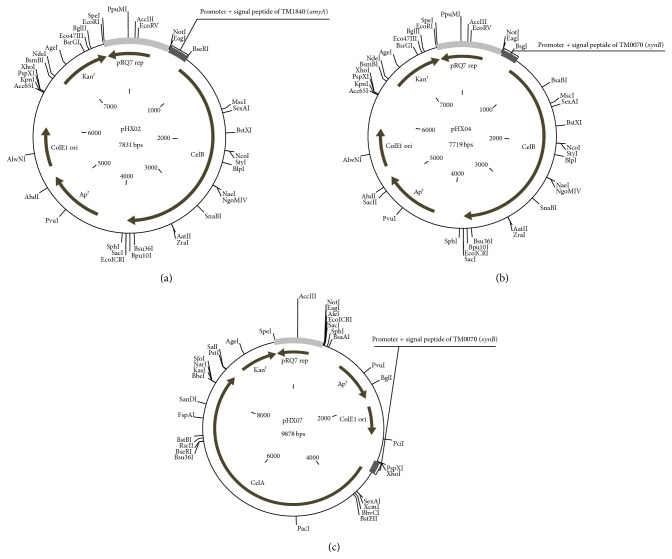
Maps of the expression vectors pHX02 (a), pHX04 (b), and pHX07 (c). Gray region represents sequence of pRQ7. Unique restriction sites are shown.

All vectors were constructed by following standard cloning methods and verified by restrictive digestions. Primers used in this study are summarized in Table 3. The* Thermotoga*-*E. coli *shuttle vector pDH10 was used as the parent vector. Inverse PCR was performed with pDH10 [23] using primers* DBs F* and* DBs R*, and the amplicon was digested with* Bsa*I followed by self-ligation to give rise to pDH26. With the same approach, pDH27 was generated based on pDH26 using primers* DNd F* and* DNd R*, and pHX01 was created from pDH27 using primers* DLZ F* and* DLZ R*. Compared to pDH10, pHX01 is 342 bp shorter and is free of the* Bsa*I and* Nde*I recognition sites, which makes it a better cloning vector than pDH10. Based on pHX01, intermediate vectors pHX02.1, pHX04.1, and pHX07.1 were constructed. Vector pHX02.1 carries the promoter and signal peptide region of* TM1840* (*amyA*), which was inserted immediately upstream of the* Ap* ^*r*^ gene (Figure 1(a)); primers* AmP F* and* Amp R* were used to amplify the desired region from* T. maritima* chromosome, and the amplicon was digested with* Not*I and* Sac*I. Vectors pHX04.1 and pHX07.1 both carry the promoter and signal peptide region of TM0070 (*xynB*), but one has the insert upstream of the* Ap* ^*r*^ gene (Figure 1(b)) and the other has it downstream of the* ori *region (Figure 1(c)). Because the two insertions sites were recognized by different restriction enzymes, primers* XyBPB F* and* XyBPB R* were used to amplify the insert for pHX04.1, and the amplicon was digested with* Not*I and* Sac*I; primers* XyBPA F* and* XyBPA R* were used to prepare the insert for pHX07.1, and the amplicon was digested by* Xho*I and* Pst*I. As a result, a* Bsa*I site was introduced immediately after the signal peptide sequence in each vector to facilitate the insertion of the coding regions of the* C. saccharolyticus* cellulases.

Once the regulation and transportation regions were in place, the cellulases genes from* C. saccharolyticus *were inserted into pHX02.1, pHX04.1, and pHX07.1 to give rise to pHX02, pHX04, and pHX07, respectively (Figure 1). The total DNA of* C. saccharolyticus* DSM 8903 was used as the template. Primers* CelB F* and* CelB R* were used to amplify Csac_1078 (*celB*), and the amplicon was digested with* Bsa*I and* Sac*I. Primers* CelA F* and* CelA R* were used to amplify Csac_1076 (*celA*), and the amplicon was digested with* Bbs*I and* Pst*I.

The transformation of* E. coli *was done with standard calcium chloride method, and the transformation of* Thermotoga* was done by natural transformation, as described previously [13]. The DNA substrates used to transform* Thermotoga* were* in vitro* methylated by methylase M.* Tne*DI [26, 27].

### 2.3. Detection of Endoglucanase Activity with CMC Plates

Endoglucanase activities were evaluated using Congo red assays [28]. For preliminary screening,* E. coli* transformants were inoculated on CMC plates (1% NaCl, 0.5% yeast extract, 0.2% CMC, 1.5% agar) and incubated at 37°C for overnight. The plates were then kept at 77°C for 8 h, stained with 0.1% Congo red (dissolved in water) at room temperature for 15 min, and washed with 1 M NaCl until clear halos showed up. After that, the plates were rinsed with 1 M HCl, which changed the background color into blue, providing a better contrast for the halos. To test liquid cultures, 40 *μ*L of each normalized overnight culture was directly loaded onto CMC plates. To minimize the sizes of the loading spots, the liquid cultures were loaded through 8 times with 5 *μ*L in each loading. The next round of loading only happened when the liquid from the previous loading had been completely absorbed. To localize the expression of the recombinant proteins, supernatants were collected from 1 mL of normalized overnight culture by centrifugation. Meanwhile, the cells were washed once with fresh medium and resuspended in 1 mL of the same medium.

### 2.4. Detection of Endoglucanase Activities with Zymogram

Native polyacrylamide gel electrophoresis was modified from a previous report [29]. SDS (sodium dodecyl sulfate) was omitted from the gel (10%, w/v), but CMC was added to a final concentration of 0.08% (w/v). Protein samples were prepared in the absence of SDS, reducing agents, and the heat treatment. After electrophoresis, gels were rinsed with deionized water for 3 times prior to immersion in 0.25 M Tris-HCl (pH 6.8) for 8 h at 77°C. Following the enzymatic reaction, gels were visualized with Congo red, as detailed above.

### 2.5. Detection of Exoglucanase Activity

MUC (4-methylumbelliferyl *β*-D-cellobioside) agar was used to detect exoglucanase activity [17, 30]. Under the hydrolysis of exoglucanase, MUC is converted to cellobiose and MU (4-methylumbelliferone), which shows fluorescence under ultraviolet light. Forty microliters of normalized overnight culture of each* Thermotoga* transformant was spotted on MUC plates, incubated at 77°C for 8 h, and examined under UV light. The formation of fluorescent halos surrounding the loading spots indicates the activity of exoglucanase.

## 3. Results and Discussion

### 3.1. Expression and Localization of the Chimeric Enzymes in* E. coli*

**Figure 2:**
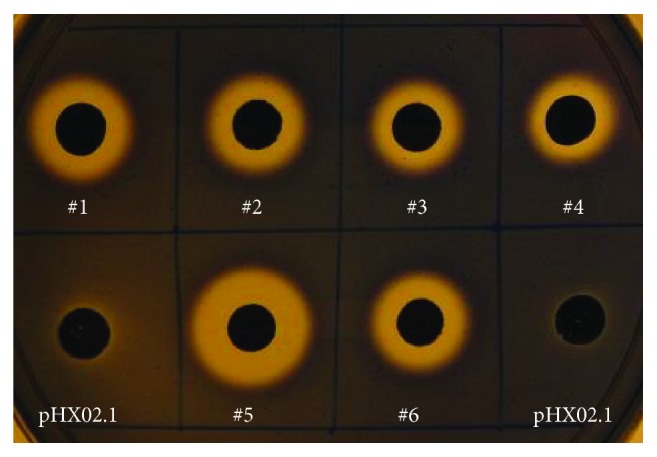
Detection of endoglucanase activities in* E. coli* DH5*α* transformants (showing DH5*α*/pHX02 as an example). #1–#6, DH5*α*/pHX02 transformants; DH5*α*/pHX02.1, negative control.

**Figure 3:**
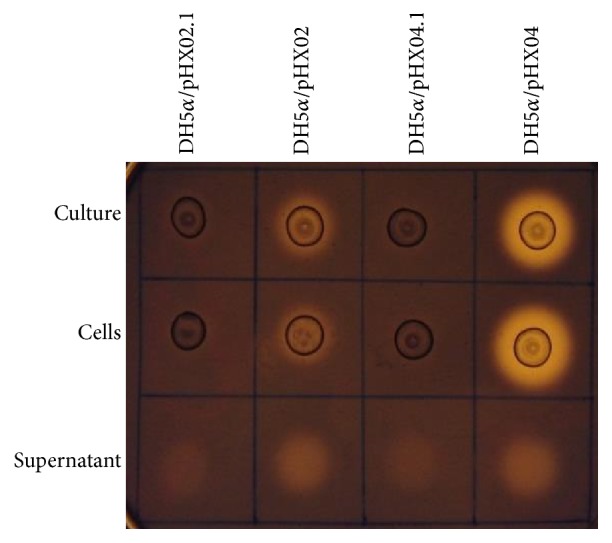
Localization of recombinant enzymes in* E. coli* DH5*α* transformants. DH5*α*/pHX02.1 and DH5*α*/pHX04.1 were used as negative controls.

In the endoglucanase activity screening experiment, all tested DH5*α*/pHX02 (Figure 2), DH5*α*/pHX04, and DH5*α*/pHX07 strains showed clear halos surrounding the overnight colonies, indicating functional expression of the recombinant cellulases in* E. coli*. To determine the localizations of the recombinant cellulases, cultures were normalized and supernatants and cell suspensions were tested separately. Because pHX07 and pHX04 share the same localization signal, experiments were carried out with just pHX04 and pHX02 transformants. On CMC plates, the pHX04 transformant demonstrated a higher endoglucanase activity than the transformants of pHX02 (Figure 3). For both constructs, most of the endoglucanase activity was associated with the cell suspensions (Figure 3). This is not surprising for pHX02, because its fusion protein is designed to be anchored on the outer membrane of a Gram-negative host. As for pHX04, since its fusion protein is meant to be released into the medium, these data suggest that the signal peptide of TM0070 (XynB) is not functional in* E. coli, *even though its promoter is.

### 3.2. Detection of Endoglucanase Activities in* Thermotoga*

**Figure 4:**
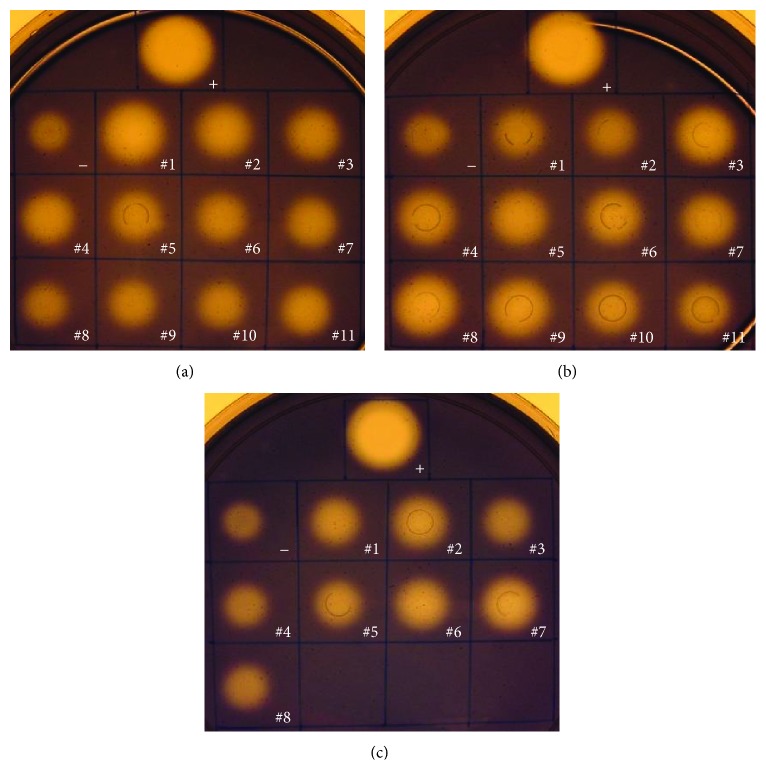
Screening of endoglucanase activities in* Thermotoga* transformants RQ2/pHX02 (a), RQ2/pHX04 (b), and RQ2/pHX07 (c). +,* C. saccharolyticus* DSM 8903, positive control; −,* T.* sp. strain RQ2, negative control.

As our main purpose is to express the recombinant cellulases in* Thermotoga*, we next transformed these 3 expression vectors into* T.* sp. strain RQ2. Eleven* T.* sp. strain RQ2/pHX02, eleven RQ2/pHX04, and eight RQ2/pHX07 transformants were isolated and tested with Congo red assays. Wild type* T.* sp. strain RQ2 and* C. saccharolyticus* DSM 8903 were used as the negative and positive controls. Almost all transformants showed enhanced endoglucanase activities compared with the wild type strain (Figure 4), indicating the successful transformation and expression of the recombinant enzymes. Next we set out to validate the transformants with PCR and restriction digestions.

### 3.3. Validating* Thermotoga* Transformants

**Figure 5:**
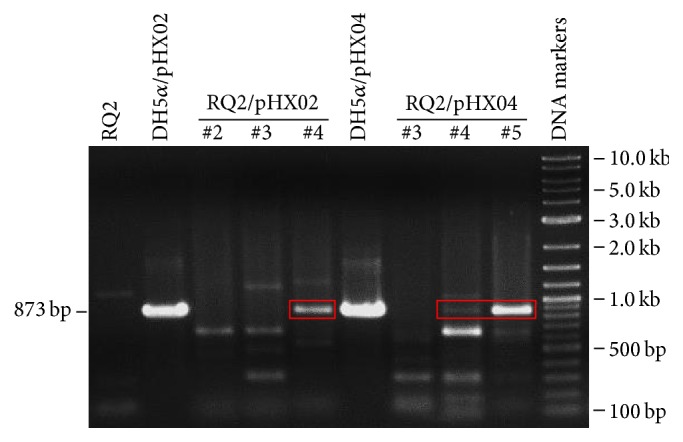
Amplification of the exoglucanase domain in* T.* sp. strain RQ2 transformants.

**Figure 6:**
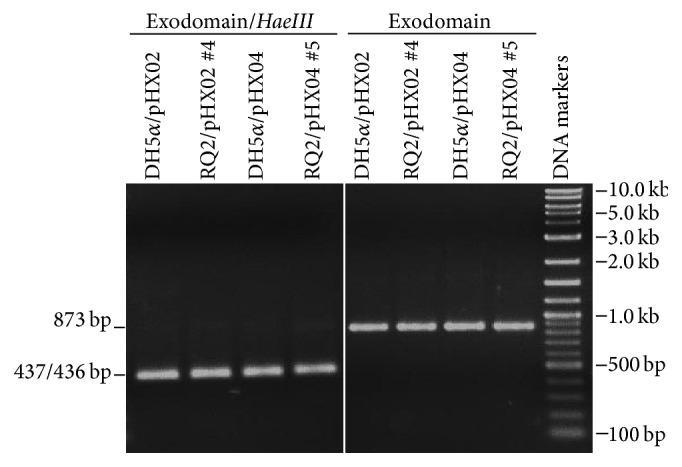
Restriction digestion of PCR products of the exoglucanase domain of* T.* sp. strain RQ2 transformants.

Three RQ2/pHX02 transformants (#2, #3, and #4) and three RQ2/pHX04 transformants (#3, #4, and #5) were picked up from SVO plates and grown overnight in liquid SVO medium with 150 *μ*g kanamycin mL^−1^. Plasmid extracts were prepared and used as the templates to amplify the exoglucanase domain of recombinant* celB* gene with primers* celBV F* and* celBV R*. One RQ2/pHX02 isolate (#4) and two RQ2/pHX04 isolates (#4 and #5) showed bands with the expected size (in addition to some nonspecific bands) (Figure 5). The positive bands from RQ2/pHX02 #4 and RQ2/pHX04 #5 were gel-purified, reamplified using the same primers, and digested with* Hae*III (Figure 6). Both amplicons developed the expected digestion profile, demonstrating the authenticity of the two transformants, which were then selected to be further characterized in later studies. The attempts to amplify either exo- or endoglucanase domain from the pHX07 transformants failed. Since pHX07 carries the* celA* gene, instead of the* celB* as found in pHX02 and pHX04, further optimization of PCR conditions and/or primer selections may eventually allow one to validate the transformants of this vector. Nevertheless, pHX07 #2 was selected for further studies, because it at least displayed strong endoglucanase activity on CMC plates.

### 3.4. Detection of the Exoglucanase Activity in* Thermotoga*

**Figure 7:**
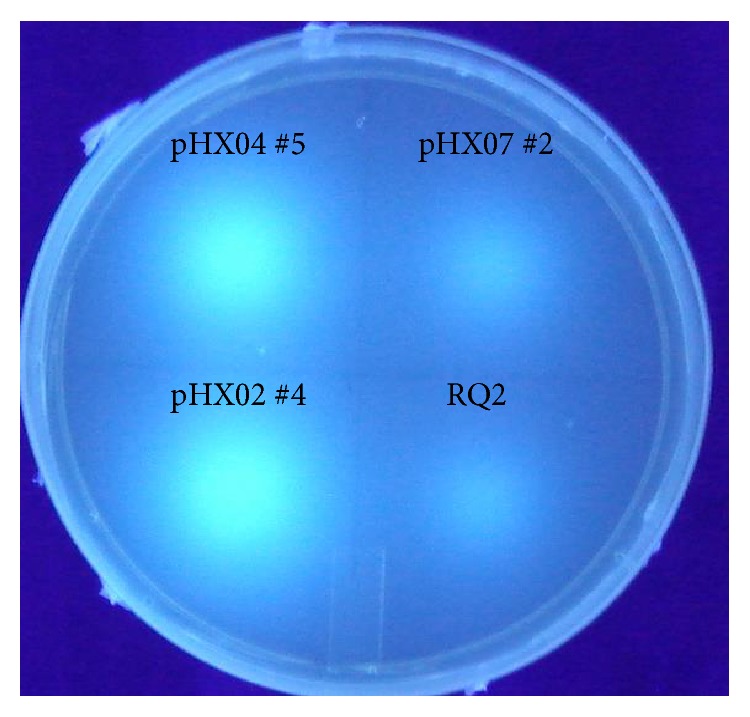
Detection of exoglucanase activities in* T.* sp. strain RQ2 transformants. RQ2, wild type strain, used as the negative control.

The exoglucanase activity of the* Thermotoga *transformants was tested with MUC plates (Figure 7). Compared to the host strain, RQ2/pHX02 and RQ2/pHX04 demonstrated greatly enhanced exoglucanase activities, as bright fluorescent light emitted under UV light from the spots where their overnight cultures were loaded. This suggests that the exoglucanase domain of* Caldicellulosiruptor* CelB was successfully expressed and fully functional in* T.* sp. strain RQ2. However, the fluorescence level displayed by RQ2/pHX07 was at about the same level to the wild type strain. Because the recombinant enzymes carried by pHX04 and pHX07 share the same promoter and signal peptide, the low level of exoglucanase activity presented by RQ2/pHX07 indicates the exodomain of* Caldicellulosiruptor* CelA was either lost (which echoes the PCR results above) or not functional in* T.* sp. strain RQ2.

### 3.5. Localization of the Recombinant Cellulases in* Thermotoga*

**Figure 8:**
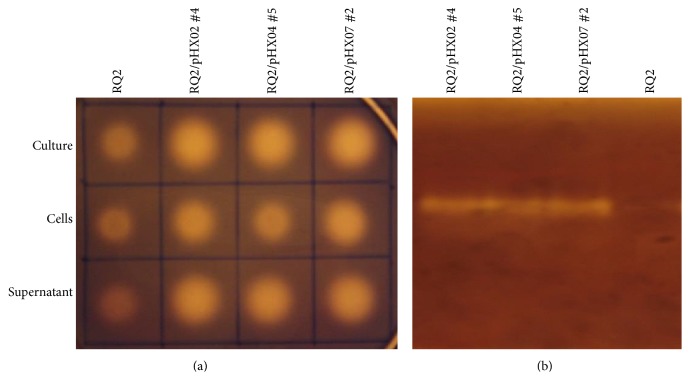
Localization of recombinant proteins in* T.* sp. strain RQ2 transformants. (a) CMC plate; (b) zymogram of the supernatants.* T. *sp. strain RQ2 was used as the negative control.

Localization of the recombinant enzymes was carried with* T.* sp. strain RQ2/pHX02 #4, pHX04 #5, and pHX07 #2 by comparing the endoglucanase activity in supernatants versus cell suspensions. Unlike what happened in* E. coli* where the majority of the activity was associated with cell suspensions, the* Thermotoga* transformants had about half of the endoglucanase activity found in supernatants (Figure 8(a)). The endoglucanase activity in the supernatants was double-checked with native polyacrylamide gels followed by Congo red assay. All supernatants showed brighter bands than the wild type strain, indicating enhanced cellulases activities (Figure 8(b)). No protein bands were detectable in the supernatants by Coomassie brilliant blue staining. The cell suspensions retained the other half of the enzymatic activities, probably because of the cytoplasmic proproteins of the chimeric enzymes (Figure 8(a)).

### 3.6. Stabilities of the Recombinant Strains

**Figure 9:**
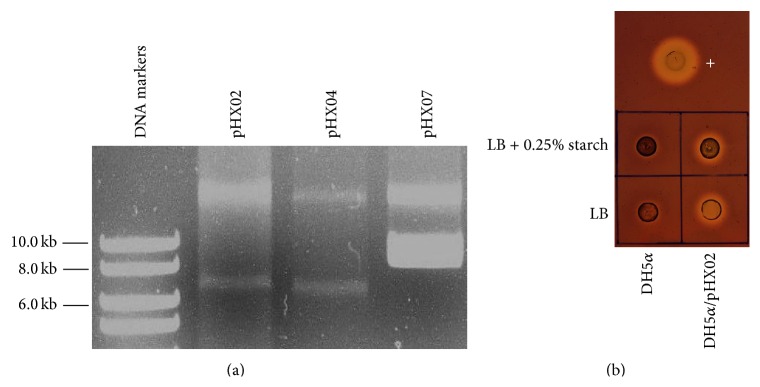
Stabilities of the* E. coli* recombinant strains. The bacterial cultures were transferred for four consecutive times with 100 *μ*g mL^−1^ ampicillin and were subject to plasmid extraction (a) and Congo red plate assays (showing DH5*α*/pHX02 as an example) (b). Samples used for plate assays were prepared from normalized cultures. +,* C. saccharolyticus *DSM 8903; LB, Luria-Bertani broth.

Stabilities of the* E. coli *and* T.* sp. strain RQ2 recombinant strains were tested by consecutively transferring corresponding cultures under the selection of antibiotics. After four transfers, all shuttle vectors were readily detected in the* E. coli *transformants (Figure 9(a)). The endo- and exodomains of* celA* and* celB* were also successfully amplified from the plasmid DNA extracts (data not shown). Congo red assays with DH5*α*/pHX02 showed that the enzyme was as active as before and the expression level of the enzyme was not affected by the inclusion of 0.25% starch in the medium [21, 25] (Figure 9(b)). These results indicate that, in* E. coli* DH5*α*, the constructed vectors are stably maintained and the enzymes are constitutively expressed.

**Figure 10:**
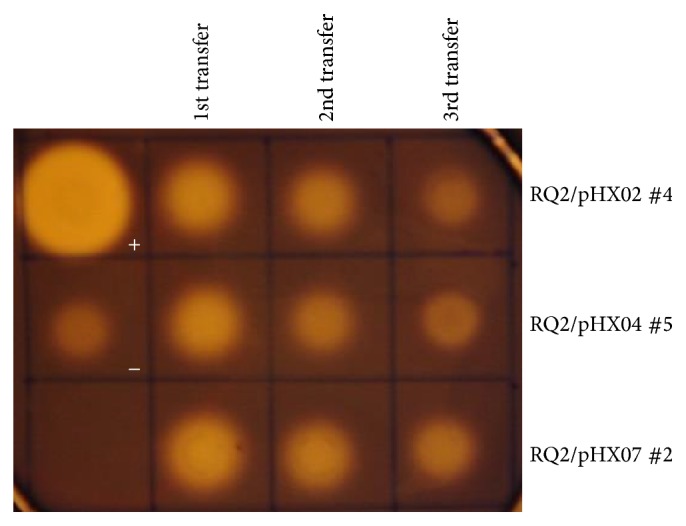
Stabilities of the* T.* sp. strain RQ2 recombinant strains. The bacterial cultures were transferred for three consecutive times with 150 *μ*g mL^−1^ kanamycin. Samples used for plate assays were prepared from normalized cultures. +,* C. saccharolyticus *DSM 8903, −,* T*. sp. strain RQ2.

Unfortunately, in* T.* sp. strain RQ2, the vectors seemed to be gradually lost, as indicated by decreasing enzyme activities with each transfer. After the 3rd transfer, the activities of both endo- (Figure 10) and exoglucanase (data not shown) were at the same level as the negative control. Trying to induce the cultures with 0.25% starch or 0.25% xylose [21, 25, 31] did not result in improved expression of the enzymes, suggesting the diminishing of enzyme activities was a result of loss of genes rather than a lack of expression. Our previous study demonstrated that the* Thermotoga-E. coli* shuttle vector pDH10 is stably maintained in both* E. coli* and* Thermotoga* [23]. However, the vectors constructed in this study, which are derived from pDH10, were only stable in* E. coli*, but not in* Thermotoga*. This might be due to different genetics of the* Thermotoga* hosts. In the previous study,* T.* sp. strain RQ7 and* T. maritima* were used, and in this study, the host was* T.* sp. strain RQ2. Cryptic miniplasmids pRQ7 and pMC24 have been found in* T.* sp. strain RQ7 and* T. maritima* [32, 33], but no natural plasmids have ever been seen in* T.* sp. strain RQ2. Plasmids pRQ7 and pMC24 are only 846 bp in length and encode just one apparent protein, which seems to play a role in plasmid replication but lacks the site-specific nuclease activity typical to a fully functional replication protein [34]. It is possible that the genomes of* T.* sp. strain RQ7 and* T. maritima* encode gene(s) essential to the replication of pRQ7-like plasmids, allowing the survival of pRQ7/pMC24-based vectors, whereas* T.* sp. strain RQ2 may lack such gene(s). As about half of the* Thermotoga* genomes encode uncharacterized proteins, finding such gene(s) requires thorough functional genomics studies and will be the future direction of our work.

## 4. Conclusions

This work demonstrated that it is possible to functionally express large heterologous proteins in* Thermotoga*. Transformed with the recombinant* Caldicellulosiruptor* cellulases,* T.* sp. strain RQ2 displayed increased endoglucanase activity with the expression of all three engineered enzymes, namely, TM1840 (AmyA)-Csac_1078 (CelB), TM0070 (XynB)-Csac_1078 (CelB), and TM0070 (XynB)-Csac_1076 (CelA). Exoglucanase activity was also improved significantly in* T.* sp. strain RQ2 transformants expressing the chimeric enzymes TM1840 (AmyA)-Csac_1078 (CelB) and TM0070 (XynB)-Csac_1078 (CelB). However, the* Thermotoga* transformants lost their recombinant genes after three consecutive transfers. This study represents an important milestone in the effort of using* Thermotoga* to produce biohydrogen directly from cellulosic biomass. Future studies should be focused on improving the stability of the transformants.

## Figures and Tables

**Table 1:** Number of predicted carbohydrate-active enzymes in *Thermotoga *genomes^*^.

Family categories	*T. *sp. strain RQ2	*T. maritima* MSB8	*T. neapolitana* DSM 4359	*T. petrophila* RKU-1	*T. naphthophila* RKU-10	*T. lettingae* TMO	*T. thermarum* DSM 5069
Glycoside Hydrolase family	52	49	49	50	40	36	32
Glycosyltransferase family	20	22	21	17	19	20	13
Polysaccharide Lyase family	1	1	0	1	0	1	0
Carbohydrate Esterase family	5	5	3	5	4	5	2
Carbohydrate-binding module family	20	17	17	15	15	4	8

Total	98	94	90	88	78	66	55

^*^Data collected from http://www.cazy.org/ on August 7, 2014.

**Table 2:** Strains and vectors used in this study.

Strain or plasmid	Description	Reference
*E. coli *		
DH5*α*	F^−^ *endA1 hsdR17 *(rk^−^, mk^+^)* supE44 thi*-*1 λ* ^−^ * recA1 gyrA96 relA1 deoR *Δ(*lacZYA*-*argF*)-U169*ϕ*80d*lacZ*ΔM15	[24]
*Thermotoga *		
*T.* sp. strain RQ2	Isolated from geothermally heated sea sediment, Ribeira Quente, Săo Miguel, Azores.	[25]
*T. maritima* MSB8	The first type strain of *Thermotoga,* isolated from geothermally heated sea sediments, Porto di Levante, Vulcano, Italy.	[25]
*Caldicellulosiruptor *		
*C. saccharolyticus *DSM 8903	Isolated from wood in the flow of geothermal spring, Taupo, New Zealand.	[14]
Plasmids		
pDH10	*Thermotoga-E. coli* shuttle vector; Ap^r^, Kan^r^^*^	[23]
pDH26	pDH10-derived, with *Bsa*I site erased; Ap^r^, Kan^r^	This study
pDH27	pDH26-derived, with *Nde*I site erased; Ap^r^, Kan^r^	This study
pHX01	pDH27-derived, having *lacZ* residual sequence removed; Ap^r^, Kan^r^	This study
pHX02.1	Promoter and signal peptide region of TM1840 (*amyA*) were inserted into the *Not*I-*Sac*I sites of pHX01; Ap^r^, Kan^r^	This study
pHX02	Coding region (without the signal peptide) of Csac_1078 (*celB*) was inserted into *Bsa*I-*Sac*I sites of pHX02.1; Ap^r^, Kan^r^	This study
pHX04.1	Promoter and signal peptide region of TM0070 (*xynB*) were inserted into the *Not*I-*Sac*I sites of pHX01; Ap^r^, Kan^r^	This study
pHX04	Coding region (without the signal peptide) of Csac_1078 (*celB*) was inserted into the *Bsa*I-*Sac*I sites of pHX04.1; Ap^r^, Kan^r^	This study
pHX07.1	Promoter and signal peptide region of TM0070 (*xynB*) were inserted into the *Xho*I-*Pst*I sites of pHX01; Ap^r^, Kan^r^	This study
pHX07	Coding region (without the signal peptide) of Csac_1076 (*celA*) was inserted into the *Bsa*I-*Pst*I sites of pHX07.1; Ap^r^, Kan^r^	This study

^*^Ap: ampicillin; Kan: kanamycin.

**Table 3:** Nucleotide sequences of primers used in this study.

Primer	Sequence and restriction sites
*DBs F *	5′ATCATGGGTCTCGCGGTATCATTGCAGCACTGGGGCCAGATGGTAAGC3′ *Bsa*I
*DBs R *	5′ATCGTCGGTCTCTACCGCGGGAACCACGCTCACCGGCTCCAGATTTATCAGC3′ *Bsa*I
*DNd F *	5′ATCGTCGGTCTCTATATG*C*ATGTGCACCAAACCACTTTGAGTACGTTCCCG3′ *Bsa*I
*DNd R *	5′ATCGTCGGTCTCCATATATTATTTAGAGGACCTTATATTCCCCAAGATTGG3′ *Bsa*I
*DLZ F *	5′GTCTGACTAGTGCAACGCATGCGAGGTTCTAGAGATTAGGGTGATGGTTCACGTAGTGG3′ *Sph*I
*DLZ R *	5′GACACGCATGCCGACGGCCAGTGAATTGTAATACGAC3^′ ^ *Sph*I
*AmP F *	5′CGAGGAACGAAGCGGCCGCGGACACCTCCTTTAGATTACAAAGAGTTTAC3′ *Not*I
*AmP R *	5′GACTTAGAGCTCGATGACGGTCTCACTGGGCTAGTACCATCTGTGTTTGTGCTGTTTG3′ * Sac*I *Bsa*I
*XyBPB F *	5′CGAGGAACGAAGCGGCCGCGAAAACTCACCTCCCTTGATTGTATG3′ *Not*I
*XyBPB R *	5′GACTTAGAGCTCGATGACGGTCTCACTGGAGAGCTGAAAACTGGAACACATCCCAAC3′ *Sac*I *Bsa*I
*XyBPA F *	5′CGAGGACTCGAGGAAAACTCACCTCCCTTGATTGTATG3′ * Xho*I
*XyBPA R *	5′GATTAGCTGCAGGATGACGGTCTCAATGGAGAGCTGAAAACTGGAACACATCCCAAC3′ *Pst*I *Bsa*I
*CelB F *	5′GACGACGGTCTCACCAGACTGGAGTATTCCAAGTTTATG3′ *Bsa*I
*CelB R *	5′GGCGCGGAGCTCTCATCAGTGATGGTGATGGTGATGTTTTGAAGCTGGAACTGGCTCAGGCTCATTATTG3^′ ^ * Sac*I
*CelA F *	5′GACTACGAAGACATCCATGGCAGGAGGCTAGGGCTGGTTC3^′ ^ *Bbs*I
*CelA R *	5′GGCGCGCTGCAGTCATCAGTGATGGTGATGGTGATGTTGATTACCGAACAGAATTTCATATGTTG3′ * Pst*I
*CelBV F *	5′AGACGCGATGGGACATATCTATCCGGTATGG3′
*CelBV R *	5′GAAGCTGGAACTGGCTCAGGCTCATTATTG3′
